# Differentiation between cerebral alveolar echinococcosis and brain metastases with radiomics combined machine learning approach

**DOI:** 10.1186/s40001-023-01550-4

**Published:** 2023-12-09

**Authors:** Yasen Yimit, Parhat Yasin, Abuduresuli Tuersun, Abudoukeyoumujiang Abulizi, Wenxiao Jia, Yunling Wang, Mayidili Nijiati

**Affiliations:** 1 Medical Imaging Center, The First People’s Hospital of Kashi (Kashgar) Prefecture, Kashi, 844000 People’s Republic of China; 2 Department of Spine Surgery, The First Affiliated Hospital of Xinjiang Medical University, Urumqi, 830054 Xinjiang China; 3 Medical Imaging Center, Xinjiang Medical University Affiliated First Hospital, Urumqi, 830054 People’s Republic of China

**Keywords:** Cerebral alveolar echinococcosis, Brain metastases, Machine learning, Radiomics, Magnetic resonance imaging

## Abstract

**Background:**

Cerebral alveolar echinococcosis (CAE) and brain metastases (BM) share similar in locations and imaging appearance. However, they require distinct treatment approaches, with CAE typically treated with chemotherapy and surgery, while BM is managed with radiotherapy and targeted therapy for the primary malignancy. Accurate diagnosis is crucial due to the divergent treatment strategies.

**Purpose:**

This study aims to evaluate the effectiveness of radiomics and machine learning techniques based on magnetic resonance imaging (MRI) to differentiate between CAE and BM.

**Methods:**

We retrospectively analyzed MRI images of 130 patients (30 CAE and 100 BM) from Xinjiang Medical University First Affiliated Hospital and The First People's Hospital of Kashi Prefecture, between January 2014 and December 2022. The dataset was divided into training (91 cases) and testing (39 cases) sets. Three dimensional tumors were segmented by radiologists from contrast-enhanced T1WI images on open resources software 3D Slicer. Features were extracted on Pyradiomics, further feature reduction was carried out using univariate analysis, correlation analysis, and least absolute shrinkage and selection operator (LASSO). Finally, we built five machine learning models, support vector machine, logistic regression, linear discrimination analysis, k-nearest neighbors classifier, and Gaussian naïve bias and evaluated their performance via several metrics including sensitivity (recall), specificity, positive predictive value (precision), negative predictive value, accuracy and the area under the curve (AUC).

**Results:**

The area under curve (AUC) of support vector classifier (SVC), linear discrimination analysis (LDA), k-nearest neighbors (KNN), and gaussian naïve bias (NB) algorithms in training (testing) sets are 0.99 (0.94), 1.00 (0.87), 0.98 (0.92), 0.97 (0.97), and 0.98 (0.93), respectively. Nested cross-validation demonstrated the robustness and generalizability of the models. Additionally, the calibration plot and decision curve analysis demonstrated the practical usefulness of these models in clinical practice, with lower bias toward different subgroups during decision-making.

**Conclusion:**

The combination of radiomics and machine learning approach based on contrast enhanced T1WI images could well distinguish CAE and BM. This approach holds promise in assisting doctors with accurate diagnosis and clinical decision-making.

**Supplementary Information:**

The online version contains supplementary material available at 10.1186/s40001-023-01550-4.

## Introduction

The hydatid disease, which includes two different diseases—cystic echinococcosis, and alveolar echinococcosis [1]. Alveolar echinococcosis is a lethal parasitic disease, its endemic area is limited to the northern hemisphere, where includes Japan, parts of China, middle Asia, Russia, parts of Iran and Türkiye, central Europe and North America. Its primary host is the red fox; however, domestic dogs play crucial role in transmission to humans. Humans get infected through ingest foods or water polluted with eggs or get in touch with contaminated soil or direct contact with canid hosts [2–6]. Liver is the initial site of mass infestation, the larva may spread to other organs by regional extension or distant metastasis through hematogenous or lymphatic pathways [7]. Cerebral alveolar echinococcosis (CAE) is a rare and severe parasitic infection that affects central nervous system, accounts for about 1% of extrahepatic involvement cases, CAE poses significant threat to patients infected by this parasite. Destructive brain lesions can result in a number of neurological disorders. There is a high risk of morbidity and death with CAE, there are many difficulties in diagnosing and treating this parasitic infection [8].

Brain metastases (BM) consist of about 50% of supratentorial brain tumors, and are the most frequently encountered type of secondary malignant brain tumor. Brain metastases are commonly seen in patients with lung, breast cancer, and melanoma [9, 10]. In daily clinical practice, it is easy to diagnose CAE and BM cases in patients with a definite history of extracerebral AE and primary malignancies. However, when clinical information is limited, or CAE is found in non-endemic areas, it has difficulty to differentiate them accurately.

Radiomics and machine learning have become increasingly popular topics in medical imaging and nuclear medicine in recent years. Generally, radiomics aims to extract a wealth of information from medical images, converting them into a plethora of minable data that are difficult to discern with the human eye, providing valuable insights into tumor physiology and phenotypes [11]. Numerous researchers have successfully utilized radiomics approaches to achieve accurate tumor differentiation and assess tumor biology [12, 13]. Machine learning leverages sophisticated algorithms to process vast amounts of data, uncovering meaningful patterns that may be challenging even for highly skilled individuals [14]. In medicine, machine learning has found extensive use, ranging from differential diagnosis of brain tumors [13, 15, 16], classification of tumor phenotypes [17], to disease onset prediction based on patient’s electronic record [18], and evaluation of tumor immune microenvironment for predicting immunotherapy efficacy [19].

CAE and BM have remarkably similar imaging appearances, making it challenging to differentiate between them using routine imaging modalities such as magnetic resonance imaging (MRI) and computed tomography (CT). Both diseases typically present as solid-enhancing lesions with irregular or nodular patterns. They may exhibit rim enhancement or heterogeneous enhancement, indicating active disease processes. Perilesional edema is commonly observed due to blood–brain barrier disruption and an associated inflammatory response. In addition, CAE and BM can occur any parts of the brain. Therefore, it is difficult for accurate diagnosis through routine imaging modalities [20–22].

MRI is currently considered as one of the most advantageous diagnostic tools for evaluating the nervous system. However, conventional MRI techniques are limited in their ability to provide detailed information beyond location, size, morphology, degree of edema surrounding the lesion, and macroscopic structural changes in the lesion such as necrosis and cystic changes. These conventional diagnostic imaging methods may not always be useful.

Due to the resemblance of imaging findings between CAE and BM, accurate diagnosis is critical as the treatment therapy and prognosis differ significantly. CAE usually treated with combination of long term antiparasitic treatment and surgical resection, BM, however, managed with a multidisciplinary approach that may include surgical resection, radiation therapy, systemic chemotherapy, or targeted therapies depending on the primary tumor [23–25]. Therefore, it is critically important for clinicians to diagnose accurately before initiating clinical intervention. Moreover, in the non-endemic area, it is truly difficult accurate diagnosis. Thus, we utilize a machine learning model combined with a radiomics approach to distinguish the two diseases.

## Methods

Our institutional review board at The First Affiliated Hospital of Xinjiang Medical University and The First People’s Hospital of Kashi Prefecture gave its approval for this study. Given that the study was retrospective, written informed permission was not required.

### Study population

**Fig. 1:**
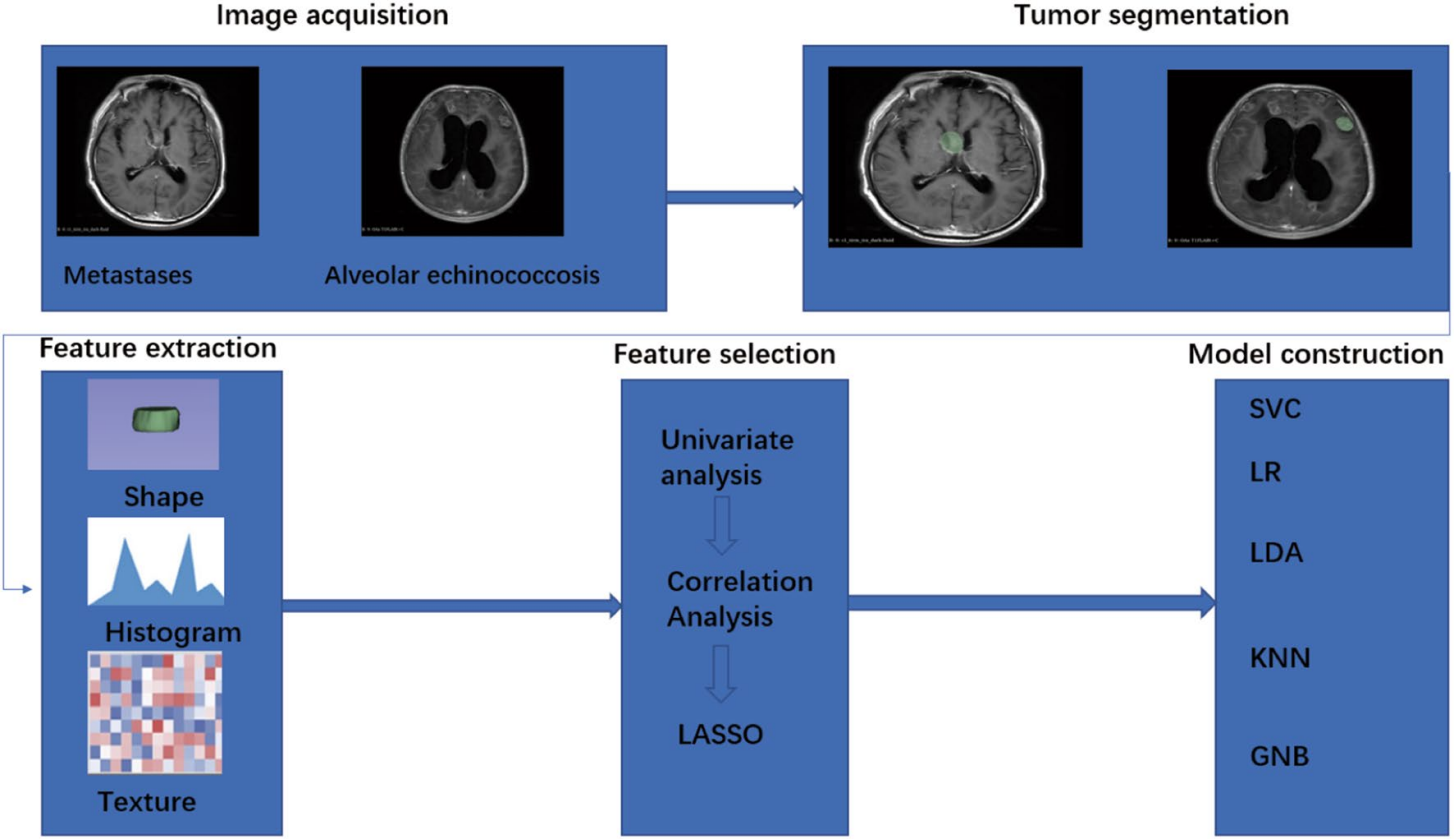
System overview of the whole research

To identify patients diagnosed with CAE and BM, we conducted a search in two hospitals from January 2014 to December 2022. Among the cases, 30 CAE cases were from Xinjiang Medical University First Affiliated Hospital, while 100 BM cases were from The First People’s Hospital of Kashi Prefecture. We turned to look at our electronic medical system. Then, 130 patients with histologically proven CAE and BM were found. The following were the inclusion criteria: (1) pathological confirmation of the CAE or BM; (2) pathologically confirmed diagnosis of hepatic alveolar echinococcosis and the clinical comprehensive diagnosis of CAE; (3) availability of T1WI, T2WI, and contrast enhanced data from preoperative multi-parametric MRI images; (4) absence of preoperative treatment history; (5) for patients with BM, have a definite history of extracerebral malignancy; (6) absence of prior brain cancer in all BM cases; and (7) availability of clinical characteristics. The following terms serve as the exclusion criteria: (1) those who had previously had treatment for CAE or BM (such as surgical, radiation, or chemotherapy); (2) patient’s imaging data are not available (3) those whose imaging artifacts made it difficult to segment lesions. All participants in this research were divided at random into a training and a testing set at the ratio of 7:3. (training set = 91, testing set = 39, in the training set CAE = 21, BM = 70, in the testing set CAE = 9, BM = 30) (Fig. 1). Considering imbalanced class data would incur the risk of biased model performance and predictions. To address this issue, we deployed the SMOTE (Synthetic Minority Over-sampling Technique) technique where it oversamples the minority class by creating synthetic samples [26].

### Imaging characteristics

Magnetic resonance imaging scanners named 3.0-T Signa Hdx MR scanner (General Electric, USA) were used. All images included axial T1WI sequence, axial T2WI sequence, axial fluid-attenuated inversion recovery-FLAIR sequence, sagittal T2WI sequences, contrast-enhanced axial, sagittal, coronal T1WI sequences. The main parameters included axial T1WI: TR = 200 ms; TE = 12 ms; slice thickness = 6 mm, DTPA-Gd injections (0.1 mmol/kg, Beijing Beilu Pharmaceutical Co., Beijing China) were used for contrast-enhanced MRI scans, parameters are as follows: TR = 200 ms; TE = 12 ms; slice thickness = 6 mm. T2WI images: TR = 3900 ms; TE = 120 ms; slice thickness = 6 mm, with the field of view [FOV] = 256 × 256 matrices). Digital Imaging and Communications in Medicine (DICOM) form was used to retrieve images from the picture archiving and communication system (PACS).

### Image segmentation

Two radiologists (Yimiti Y and Tuersun A, each having more than three or more than five years of expertise in neuroradiology) independently and blindly reviewed the images on all sequences (T1WI, T2WI, FLAIR, CE images) without knowledge of the clinical data. They used 3D slicer 4.10.1 (https://www.slicer.org), they draw the three-dimensional (3D) volume of interest (VOI) of each patient on axial contrast enhanced T1WI sequence layer by layer (3D volume of tumors segmented were provided in Additional file 1). VOIs, or contrast-enhancement tumors, were identified automatically by two neuroradiologists through the use of a straightforward region-growing segmentation technique that was integrated into 3D Slicer. Afterwards, the feature results extracted by the two readers were evaluated for consistency using ICC. Features with ICC value greater than 0.8 were retained.

### Radiomics feature pre-processing and extraction

We used PyRadiomics package (version 3.0.1) to calculate all radiomics features [27]. Image features can be categorized into three groups: shape features, first-order (distribution) features, and texture features. All intensities within the VOI of MR images were discretized to 25 bins. We set the resampling parameter to 1 × 1 × 1 mm^3^ and the normalization parameter as true for the MR image before feature extraction. First, metrics like volume and surface as well as more complex variables like compactness and sphericity were determined by the segmentation’s shape. The distribution of intensities in the volume of interest was examined to produce the second category of features. These features include conventional distributional measures like the mean, median, and interquartile range as well as shape descriptors like skewness and information-theoretical metrics like entropy. Third, texture features were extracted from the volume of interest using discretized gray values. To describe patterns in the discretized gray values, various matrices were developed, including the gray-level size-zone matrix (GLSZM), gray-level run-length matrix (GLRLM), and gray-level co-occurrence matrix (GLCM). Gray-level dependence matrix (GLDM) and neighboring gray-tone difference matrix (NGTDM) are two more matrices that examined the immediate vicinity of pixels. In addition to extracting the features mentioned above, filters were applied to these images to decrease the noise that is inherent to each MR measurement. For each patient, a total of 1584 radiomics features were extracted.

### Feature selection

Due to the expectation that several attributes would be associated, such as when employing many filters on the same image. It becomes challenging for us to visualize and analyze a machine-learning model when using datasets with a large number of features. Additionally, it takes a lot of time and memory, which increases the time and spatial complexity of the model. Because of the useless features in the dataset, the model may occasionally perform poorly on the testing data. Consequently, to reduce the number of features needed for training, feature-selection algorithms were taken into consideration. Univariate analysis -were conducted for normal distributed features using *t*-test while others were *Mann–Whitney U* tests—removed radiomics features with not significant difference between two groups. (*P* > 0.05) Then *Pearson* correlation analysis was used to remove redundant and highly correlated variables. Last, the least absolute shrinkage and selection operator (LASSO) was carried out with optimal lambda to shrink unimportant feature coeffects to zero.

### Model construction and optimization

We bring our selected features to several models including Logistic regression (LR), Support vector machine classifier (SVC), k-nearest neighbors (KNN), Linear discrimination analysis (LDA) and Gaussian naive Bayes (NB) algorithms in the current study.

Model optimization is to modify the value of the various intrinsic parameters of algorithms. Any changes to any parameters may incur the prediction performance improvement or decline. Moreover, the vital procedure in the tuning process is to validate the model with tuned parameters. Yet, it is also a process with the risk of data leakage. Thus, we used grid search with nested resampling method to solve the mentioned issue when optimizing parameters, where inner resampling (cv = 3) is responsible for the tuning while outer (cv = 5) for validate the result [28]. Nested cross validation is a technique used to evaluate the performance of a machine learning model. It is a type of cross-validation where the data is split into two sets: a training set and a testing set. The training set is then further split into two sets: a validation set and a training set. The model is then trained on the training set and evaluated on the validation set. Finally, the model is tested on the testing set to evaluate its performance. This technique is useful for assessing the accuracy of a model and for selecting the best model for a given dataset [29]. Furthermore, the two kinds of strategy were compared to each other to assess the data leakage impact.

### Model evaluation

Summary statistics were calculated for the model performance, including sensitivity (recall), specificity, positive predictive value (precision), negative predictive value, accuracy, and the area under the curve (AUC). Receiver operating characteristic (ROC) was constructed. To evaluate the consistency between predicted values and actual labels, a calibration plot was created.

### Statistical analysis

To determine whether continuous features are normal, we applied the Shapiro–Wilk test. Continues features normal distribution is displayed as mean values ± standard deviation (SD) and examined via Student’s *t*-test, while the rank sum test is used to analyze non-normal distributions and expressed as interquartile range M (P75, P25). Categorical data are displayed as frequency (percentage), and Fisher’s exact or the *χ*^2^ test was used to compare the two groups. The independence of the selected features was examined using the Pearson correlation coefficients. The above statistical analysis was performed using R 4.2.2 and SPSS 25.0 software.

## Results

### Patient characteristics

Table 1 we collected 130 patients in this study. 30 CAE patients and 100 BM patients. CAE and BM groups included the following clinical characteristics: age, BMI and gender. As is seen in Table 1, no significant differences were found between age and BMI in both CAE and BM cohort; however, significant statistical difference was found in gender, CAE was more common among male in our study.

**Table 1:** Baseline of patients

Characteristics	ALL (*n* = 130) *M* (*P*_25_,*P*_75_), *n* (%)	BM (*n* = 100) *M* (*P*_25_,*P*_75_), *n* (%)	CAE (*n* = 30) *M* (*P*_25_,*P*_75_), *n* (%)	*H*/χ^2^	*P*
Age	43.50 (33.00,52.00)	43.50 (33.25,54.00)	43.00 (31.00,50.00)	− 1.515	0.130
BMI	20.00 (19.00,22.25)	20.00 (19.00,22.00)	20.00 (19.00,23.00)	0.202	0.840
Gender				14.625	< 0.001
Male	78 (60.00)	51 (51.00)	27 (90.00)		
Female	52 (40.00)	49 (49.00)	3 (10.00)		

This Table shows the detailed patient clinical characteristics of the study cohort

As is seen in this table, no significant differences were found between age and BMI in both CAE and BM cohort; however, CAE were more common among male, with significant statistical difference

### Extraction and selection of features

**Fig. 2:**
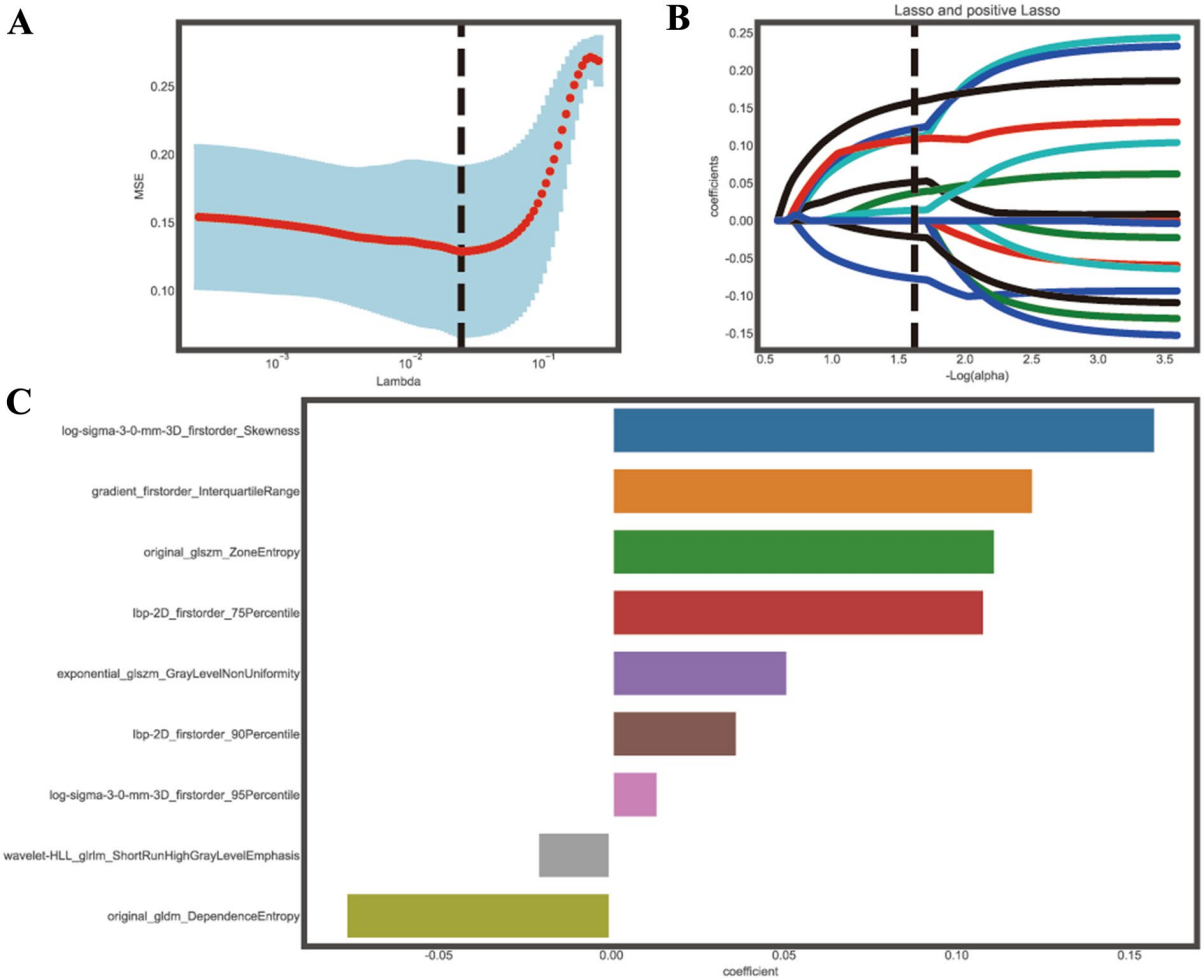
**A** LASSO regression was used to select radiomics features that could potentially distinguish between CAE and BM. Tuning of LASSO regression parameters was performed. **B** An analysis of LASSO coefficients was conducted for the 1584 radiomics features, and 9 non-zero coefficients were selected. **C** 9 valuable features were selected using the LASSO algorithm.

**Fig. 3:**
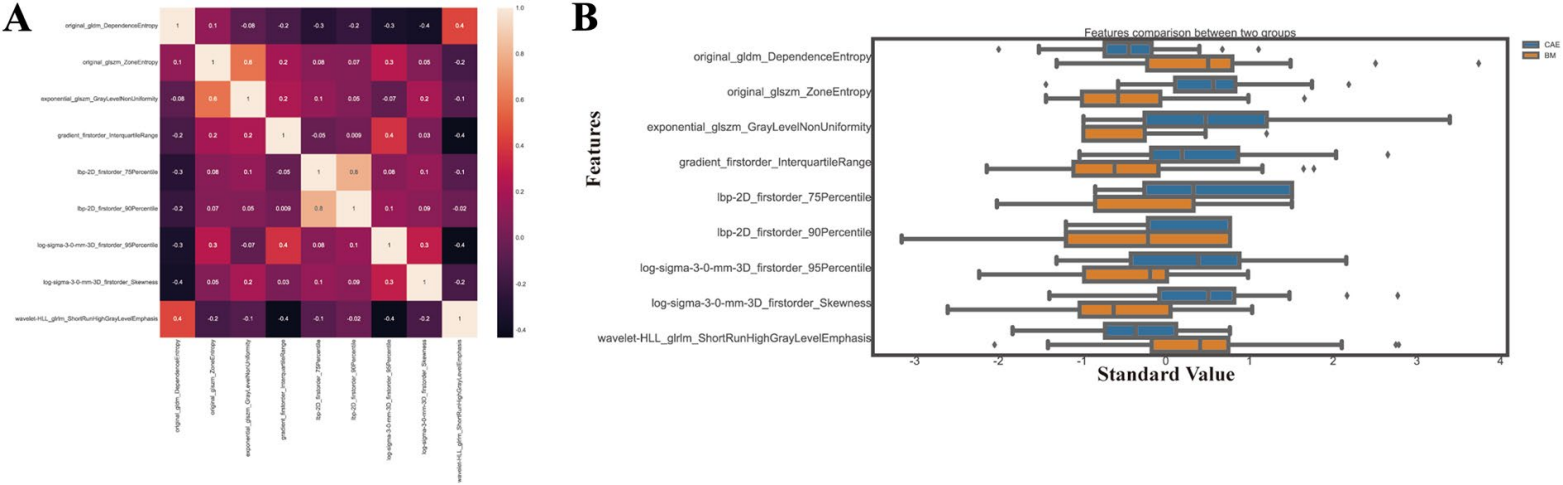
Correlation coefficients (**B**) and box plot (**A**) of the standardized value selected features

For feature selection, 127 out of 1584 features were initially screened using univariate analysis. Afterward, 26 features were selected after removing redundant variables with using highly correlated coefficients. Eventually, 9 optimal features were selected with the LASSO algorithm (Fig. 2). The Pearson correlation coefficient was used to determine whether these features were correlated. According to the results, the majority of the features were independent. The heat map of correlation among the radiomics features is displayed in (Fig. 3).

### Model optimization

**Fig. 4:**
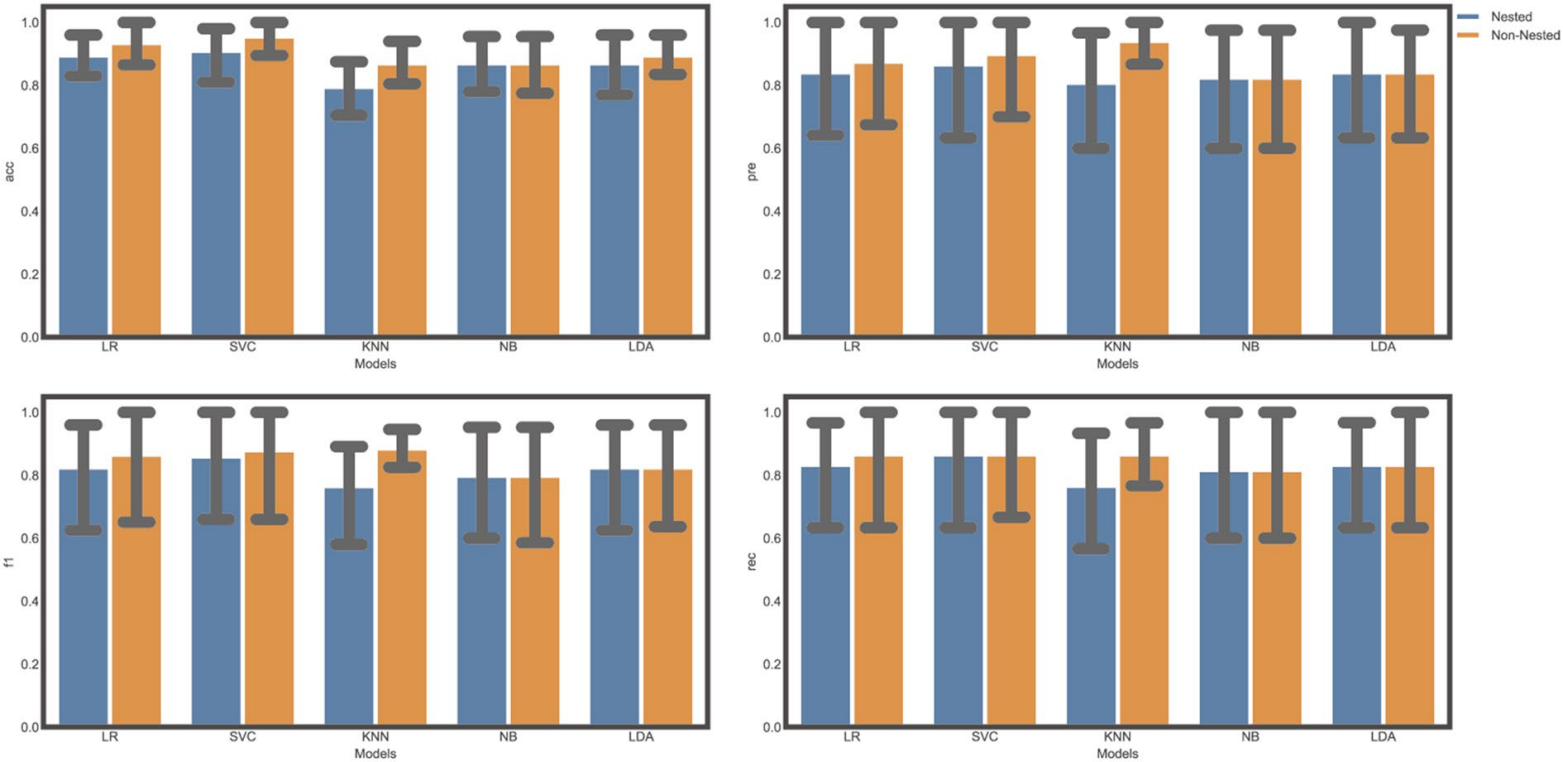
The evaluation of two resampling methods (nested or not) using various measures. **A** Are under the curve (AUC) value. **B** Precision value. **C** F1 score value. **D** Recall value

We adjusted the parameters of each model first before building the model with the entire training dataset. The comparison of nested and non-nested resampling is shown in Fig. 4. It is seen that the non-nested method showed better performance as a result of data leakage when tuning the parameters.

### Model performance evaluation

**Fig. 5:**
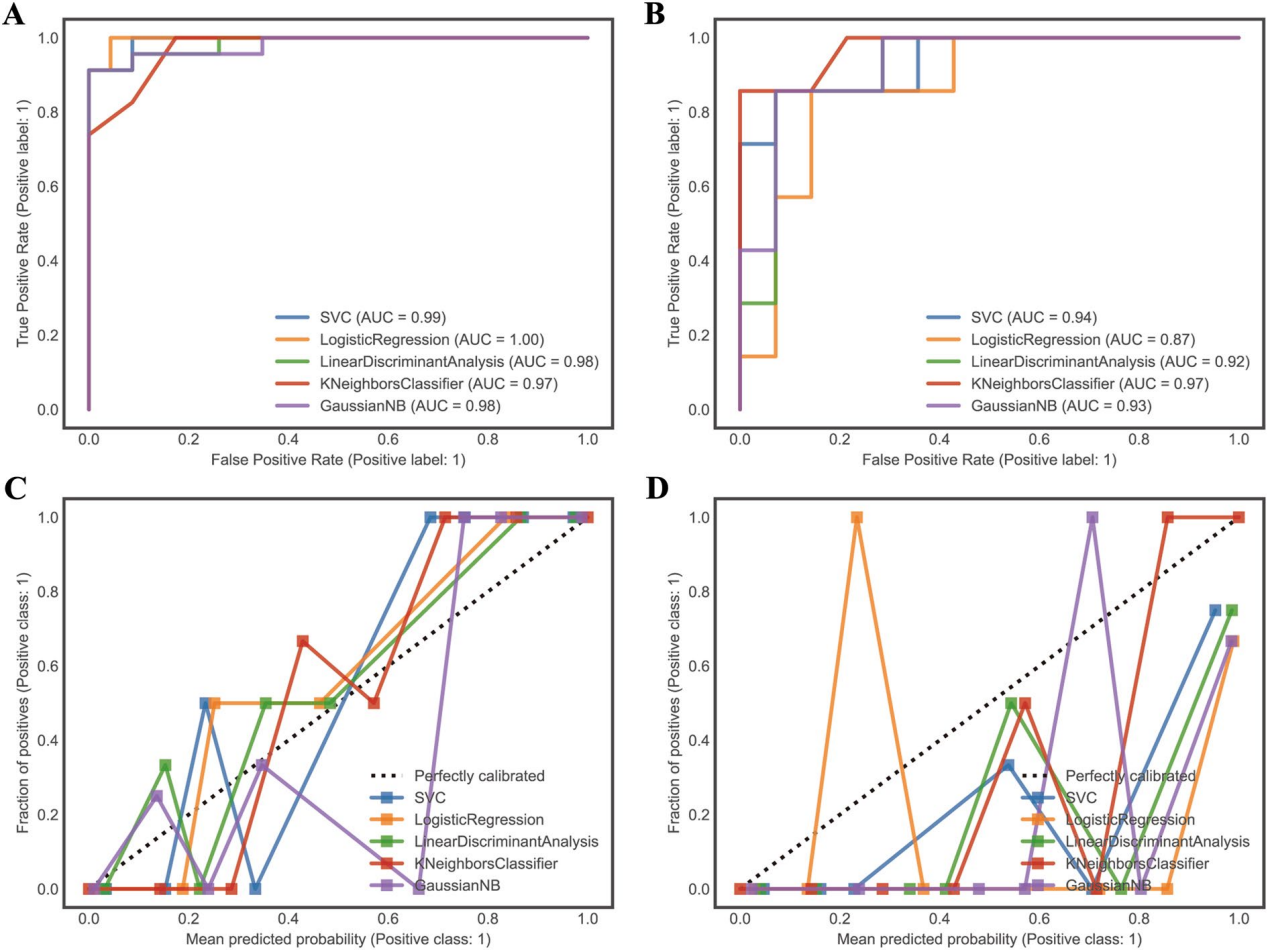
The ROC curves and calibration curves of the training set and testing set. The calibration and receiver-operating characteristic (ROC) curves of the scoring system. **A** The training set’s ROC curve. **B** The training set's calibration curve. **C** The testing set’s ROC curve. **D** The validation set's calibration curve

**Fig. 6:**
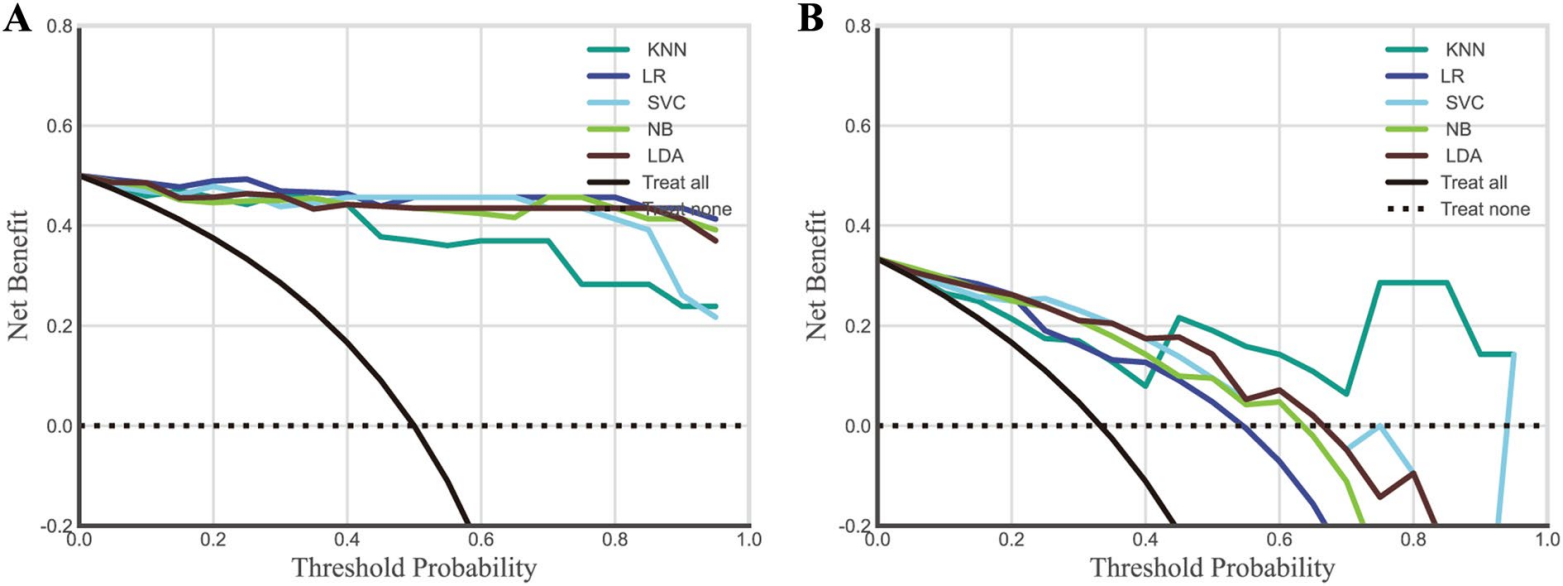
Decision curve analysis for the personalized treatment option. **A** training set, **B** testing set

The ROC curves of the five radiomics models are shown in Fig. 5A, B. The AUC of SVC, LR, LDA, KNN, and NB algorithms in training (testing) sets are 0.99 (0.94), 1.00 (0.87), 0.98 (0.92), 0.97 (0.97), and 0.98 (0.93) respectively. Other metrics are shown in Table 2. The calibration plot in Fig. 5C, D revealed the predicted and actual labels. The five radiomics models’ decision curves demonstrated that each model performs better than both the treat-all-patients and the treat-none measures in terms of result prediction (Fig. 6A, B).

**Table 2:** Model performance

Classifier	Brier loss	Log loss	Acc.	Recall	F1	Sen.	Spe.	Npv.	Ppv.
LDA	0.160	0.601	0.810	1.000	0.778	1.000	0.714	1.000	0.636
LR	0.231	1.091	0.714	0.857	0.667	0.857	0.643	0.900	0.545
SVC	0.159	0.507	0.762	0.857	0.706	0.857	0.714	0.909	0.600
KNN	0.130	0.396	0.857	1.000	0.824	1.000	0.786	1.000	0.700
NB	0.199	0.751	0.762	1.000	0.737	1.000	0.643	1.000	0.583

LDA, linear disclination analysis; LR, logistic regression; SVC, support vector classifier; KNN, K-nearest neighbors; NB; Gaussian naïve bayes; Acc., accuracy; Sen., sensitivity; Spe., specificity; Npv., negative predictive value; Ppv., positive predictive value

## Discussion

Existing literature on CAE has primarily consisted of case reports [30], lacking comprehensive studies. In this research, we conducted the most extensive systematic study to date on CAE, including 30 cases, with a significant sample size: Treatment strategies differ for CAE and BM. Radiation therapy, long-term antiparasitic medication, and surgical resection is typically performed for CAE. Conversely, BM are frequently treated using a multidisciplinary strategy that, depending on the initial tumor origin, may include radiation therapy, systemic chemotherapy, targeted medicines, or surgical resection. BM is commonly associated with advanced stages of cancer and typically carries a poor prognosis. In contrast, CAE progresses slowly and chronically, its prognosis can be greatly enhanced by prompt diagnosis and treatment. Accurate diagnoses are crucial as they can help avoid unnecessary interventions, particularly for CAE, where performing a biopsy poses a risk of parasite spillage and dissemination within the brain. However, CAE and BM share similar symptoms and imaging presentations, including neurological symptoms like seizures, headaches, focal neurological deficits, and mental disorders. Both diseases can manifest as multiple solid enhancing masses with surrounding edema on imaging examinations, posing challenges for physicians and radiologists in achieving accurate diagnoses [23–25]. Therefore, it is of great importance to accurate diagnose CAE and BM.

In this study, we aimed to develop a precise and reproducible classifier to differentiate between patients with BM and those with CAE using a wide range of radiomics features and machine learning methods. Specifically, we built five different machine learning models to accurately distinguish between CAE and BM based on conventional contrast-enhanced T1WI images. Among the models, the KNN classifier demonstrated the highest performance, with an AUC value of 0.97. It achieved a precision of 0.70, accuracy of 0.86, sensitivity of 1.0, and specificity of 0.78. On the other hand, the logistic regression algorithm displayed the lowest performance, with an AUC of 0.87, precision of 0.55, accuracy of 0.71, sensitivity of 0.86, and specificity of 0.64.

Radiomics aims to extract high-throughput quantitative image features from radiographic images and train a prediction model [31]. Since its first introduction by Philippe Lambin in 2012, radiomics has demonstrated considerable promise in developing models that can distinguish different types of tumors based on the numerous image features extracted from MRI that represent tumor heterogeneity [13, 15, 32, 33]. Radiomics combined with a machine learning approach has been widely studied in recent years. In our research, 9 valuable features were selected, which include 2 features based on log-sigma transformed images, 3 first-order features, 2 GLSZM features, 1 GLDM feature and 1 wavelet HLL feature.

As a representation of the local image structures at multiple scales, Log-sigma transformed features enable the analysis and description of complex structures, edges, and textures. The log-sigma transformation convolves the image with a sequence of Gaussian filters at various standard deviation (sigma) values to improve edges, boundaries, and other important image properties. In our research 2 valuable features were log-sigma features [34].

First-order features usually describe basic statistical or histogram-based characteristics of the data distribution, such as mean, median, standard deviation, range, skewness, kurtosis, or other statistical moments. To investigate whether CT-based texture analysis could early predict tumor recurrence from radiation-induced lung injury, Mattonen SA et al. [35] conducted a study, results showed that first-order features (energy, and entropy) achieved AUCs of 0.79–0.81 using a linear classifier. On two-fold cross validation, first-order texture reached 73% accuracy, which is similar to our research.

Spatial relationship and distribution of gray-level intensity patterns are characterized by gray-level size-zone matrix (GLSZM) features. To investigate whether peritumoral edema heterogeneity could predict glioblastoma recurrence, Long H et al. [36] have conducted MRI-based radiomics research, the results showed two GLSZM features (small area emphasis and low gray level emphasis) are among the valuable features could predict glioblastoma recurrence, which is in line with our study.

The number of patterns made up of linked voxels with comparable intensities is counted using the Gray Level Dependence Matrix (GLDM). Higher values in the dependence variance of GLDM indicate more diverse patterns in an image. In their study Peng S et al. [37] to predict neoadjuvant therapy response in breast cancer based on multi-phase contrast enhance MRI, results showed GLDM features in phase 1, 3 and 4 were valuable predictors, which is similar with our findings.

Using a series of wavelet functions that transition from higher frequency wavelets to lower frequency ones, wavelet decomposition divides up image data. The high-pass filter captures the more subtle information that is approximated by the higher frequency wavelet function, while the low-pass filter captures the remaining information that can be further deconstructed using lower frequency wavelet functions. Many researchers have found the importance of wavelet-HLL features in radiomics studies, one wavelet-HLL feature showed value in our study.

The use of radiomics-based machine learning for the diagnosis of CAE and brain metastases has several advantages over traditional methods. First, radiomics-based machine learning can provide more accurate and reliable results than traditional methods. This is because radiomics-based machine learning can extract more detailed information from medical images than traditional methods. Additionally, radiomics-based machine learning can be used to detect subtle differences between CAE and BM that may not be visible to the naked eye. Cerebral alveolar echinococcosis is a rare parasitic disease, but it is still a severe public health issue in many parts of the world. We believe that radiomics-based machine learning is a novel tool to investigate this disease, which have been proved as a powerful approach in other fields [38–41].

Due to rarity and limited data for CE, in our research we have utilized nested cross validation—when the dataset is small and there are numerous hyperparameters to adjust for the model, it is extremely helpful [42]. Nested cross-validation’s generalization ability can be deemed beneficial for a number of reasons. First off, by giving more accurate predictions of the model’s performance, it helps to reduce the risk of overfitting. The outer loop offers an objective assessment of how well the model will function on unobserved data by splitting the data into an outer and inner loop. The model is adjusted for better generalization rather than overfitting to the training data using the inner loop, which is used for hyperparameter adjustment. Secondly, the use of cross-validation helps to reduce the dependency of the performance estimate on a particular train-test split. By repeating the process multiple times, with different splits of the data, the variability in the performance estimate can be assessed. This helps to capture the model’s ability to perform well on unseen data from different perspectives, enhancing its generalization capability. Nested cross-validation also makes the model selection process more reliable. It makes it possible to compare various models or hyperparameter combinations objectively and choose the one that performs the best. This selection procedure aids in finding models that are effective on training data as well as those that generalize well to fresh, unexplored data [28, 43].

For the selection of biomarkers in high-dimensional data, the variable selection compression estimation method- LASSO has been widely used [44]. By developing a penalty function, it builds a more refined model by compressing certain coefficients while leaving others at zero. In this method, feature screening (dimension reduction) and over-fitting are both avoided during model training. In our study LR and KNN showed the best performances in training and testing sets, which is similar with previous studies [45]. These features allowed the LASSO regression model and LR, KNN classifiers to work together flawlessly in the radiomics investigation. Additionally, the LASSO algorithm chose the observed radiomics features from a variety of filters and feature classes, which shows that multiple feature categories may provide complimentary information in differentiating between the CAE and BM. Even though the biological activity underlying these radiomics features is not yet known, we hypothesize that they may be able to capture the fine radiomics qualities of microstructure and the tumor’s immediate surroundings.

Finally, radiomics combined machine learning approach has the potential to revolutionize the way we diagnose and differentiate between cerebral alveolar echinococcosis and brain metastases. Radiomics is a branch of medical imaging that uses advanced algorithms to extract quantitative features from medical images. These features can then be used to create predictive models that can accurately differentiate between CAE and brain metastases^.^

## Limitations

To the best of our knowledge, this is the first study that has used a combination of radiomics and machine learning algorithms to differentiate CAE and BM, in addition, this study includes the largest CAE cases. However, there are some limitations our study: First, due to the rarity of CAE, even though data for CAE and BM have been collected for over ten years, there is still a small sample in this study. We intend to do multicenter research in the future to address this issue. Second, since the borders of CAE and BM are more well-defined in contrast enhanced sequences than in T2WI sequences, only contrast enhanced MRI sequences were used in our study. By including multi-model imaging data in the future, our model can be improved.

## Conclusion

In conclusion, with good predicted accuracy and stability, the presented radiomics machine-learning classifier provides a non-invasive way to identify MET from GBM before surgery. We think merging radiomics analysis with machine learning techniques can enhance oncology accuracy and clinical practice.

### Supplementary Information

**Additional file 1. In the open-source software 3D Slicer, the T1-weighted axial enhanced MRI sequence is contoured layer by layer along the tumor outline, then the software automatically generates a 3D model of the tumor.**

## Data Availability

The datasets manipulated or generated in our research are available from the corresponding author upon reasonable request.

## Code availability

The code used in our research are available from the corresponding author upon reasonable request.
