# Supplementary figures and images for: Differentiation between cerebral alveolar echinococcosis and brain metastases with radiomics combined machine learning approach
Source: Eur J Med Res. 2023 Dec 9;28:577. doi: 10.1186/s40001-023-01550-4 (PMC10709961; doi:10.1186/s40001-023-01550-4)

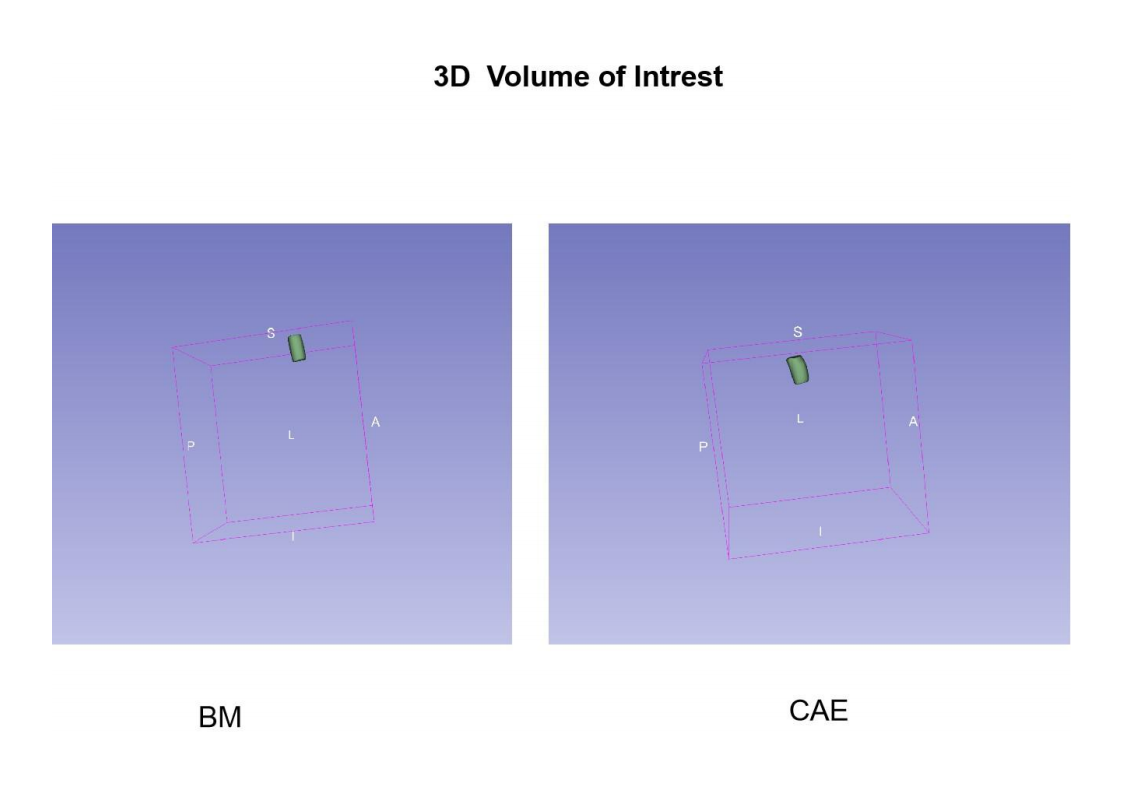

Supplement: Supplementary file 1 — Additional file 1. In the open-source software 3D Slicer, the T1-weighted axial enhanced MRI sequence is contoured layer by layer along the tumor outline, then the software automatically generates a 3D model of the tumor. [file 40001_2023_1550_MOESM1_ESM.docx]
